# Endoplasmic Reticulum Stress and Associated ROS

**DOI:** 10.3390/ijms17030327

**Published:** 2016-03-02

**Authors:** Hafiz Maher Ali Zeeshan, Geum Hwa Lee, Hyung-Ryong Kim, Han-Jung Chae

**Affiliations:** 1 Department of Pharmacology and New Drug Development Institute, School of Medicine, Chonbuk National University, Jeonju, Chonbuk 561-180, Korea; hmaherali@gmail.com (H.M.A.Z.); heloin@jbnu.ac.kr (G.H.L.); 2 Department of Dental Pharmacology and Wonkwang Biomaterial Implant Research Institute, School of Dentistry, Wonkwang University, Iksan, Chonbuk 570-749, Korea

**Keywords:** ER stress, reactive oxygen species, Nox4, glutathione, NADPH-dependent p450 reductase, calcium

## Abstract

The endoplasmic reticulum (ER) is a fascinating network of tubules through which secretory and transmembrane proteins enter unfolded and exit as either folded or misfolded proteins, after which they are directed either toward other organelles or to degradation, respectively. The ER redox environment dictates the fate of entering proteins, and the level of redox signaling mediators modulates the level of reactive oxygen species (ROS). Accumulating evidence suggests the interrelation of ER stress and ROS with redox signaling mediators such as protein disulfide isomerase (PDI)-endoplasmic reticulum oxidoreductin (ERO)-1, glutathione (GSH)/glutathione disuphide (GSSG), NADPH oxidase 4 (Nox4), NADPH-P450 reductase (NPR), and calcium. Here, we reviewed persistent ER stress and protein misfolding-initiated ROS cascades and their significant roles in the pathogenesis of multiple human disorders, including neurodegenerative diseases, diabetes mellitus, atherosclerosis, inflammation, ischemia, and kidney and liver diseases.

## 1. Introduction

The endoplasmic reticulum (ER) is a fine network of tubules that performs many versatile functions in the cell. The ER is the primary organelle for secretory pathways in all eukaryotic cells [1,2]. It is also responsible for protein folding, biosynthesis, translocation, and post-translational modifications including glycosylation, disulfide bond formation, and chaperone-mediated protein folding processes [2,3]. Before being targeted to the appropriate organelle, trans-membrane proteins and secretory proteins enter the ER to undergo the folding process. The proteins that do not fold properly within the specified period of time are targeted for ER-associated degradation (ERAD) via the ubiquitin-proteasome system [4]. To promote protein folding and prevent aggregation, a number of chaperones and enzymes work together to limit the workload through ERAD [5]. One of the best-characterized and most abundant chaperones in the ER protein folding machinery is immunoglobulin heavy chain binding protein/glucose regulated protein 78 (BiP/Grp 78), which directs misfolded proteins toward ERAD. When there is an increase in workload, proteins cannot reach their native folding state, leading to aggregation of unfolded proteins in the ER lumen; this condition is called ER stress and can deleteriously affect cell homeostasis [6]. To restore cell homeostasis after ER stress, a sequence of reactions known as the unfolded protein response (UPR) is initiated [7,8].

Under prolonged and severe ER stress, the UPR can become cytotoxic (including apoptosis) rather than cytoprotective. Among the UPR signaling pathways, there are three predominant and unique signaling transduction mechanisms: inositol-requiring enzyme 1α (IRE1α), protein kinase RNA (PKR)-like kinase (PERK), and activating transcription factor 6 (ATF6). These mechanisms can detect unusual conditions in the ER lumen and transmit signals to the cytosol, which are then directed toward the nucleus by transcription factors [9]. After activation, each mechanism induces downstream responses.

IRE1α is a transmembrane protein responsible for protein kinase and endoribonuclease activities; it also controls its own expression [10,11]. It has a cytosolic RNAse domain that excises an intron from mRNA to augment the folding capacity of the ER by generating a potent transcription activator X-box binding protein 1 (XBP1). To reduce the entry of additional unfolded proteins, the active RNAase can also cleave ER-localized messages leading, to degradation [12,13,14]. Through XBP1 activation, the UPR and ERAD restore homeostasis and thereby cytoprotection. IRE1α also activates apoptotic signaling kinase-1 (ASK-1), activating downstream factors such as Jun-N-terminal kinase (JNK) and p38 mitogen-activated protein kinase (p38 MAPK), which enhance apoptosis [15,16,17].

ATF6, a type II transmembrane protein, is another sensor of ER stress, up-regulating chaperones, and components of the ERAD pathway [18]. This protein moves from the ER to the Golgi; proteolysis then sequentially occurs by site-1 and site-2 proteases (S1P and S2P), leading to the release of its amino terminal transcription factor domain from the membrane [19]. In addition, activation transcription factor (ATF)/cAMP response elements (CRE) and ER response elements (ERSE-1) can trigger the objective genes, *Bip/Grp78*, *Grp94*, and C/EBP homology protein (CHOP) [20].

PERK is also responsible for decreasing the workload by inhibiting mRNA translation under ER stress and preventing further synthesis and thus protein folding. PERK phosphorylates eukaryotic initiation factor 2 (eIF2α) to reduce the GTP-bound form (initial phase of polypeptide synthesis) [21] and allow translation of ATF4, one of the UPR-dependent signaling proteins. Other activation mechanisms involve IRE1α-dependent ASK and p38 MAPK activation, which further activate CHOP to contribute to reactive oxygen species (ROS) generation [22]. Herein, we discuss persistent ER stress and protein misfolding that initiate ROS cascades and are known to play significant roles in disease pathogenesis. How can ROS be generated by the various ER pathways? What is the role of calcium in ROS production? The roles of redox mediators, which cause multiple human disorders, including neurodegenerative diseases, diabetes mellitus, atherosclerosis, inflammation, ischemia, and kidney and liver diseases, will be discussed below.

## 2. How Is Reactive Oxygen Species (ROS) Induced through Endoplasmic Reticulum (ER) Stress?

### The Specific Mechanism of ER Stress-Induced ROS during the ER Folding Process

**Figure 1:**
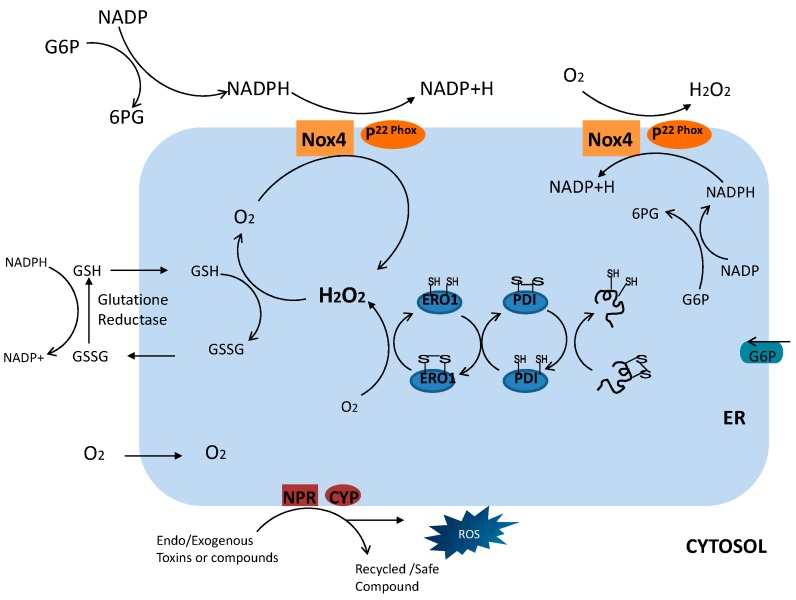
Depiction of endoplasmic reticulum (ER) redox communication by mediators. During the protein folding process, production of reactive oxygen species (ROS) by possible surrounding sources, such as NADPH oxidase 4 (Nox4), NADPH-P450 reductase (NPR), and GSH topologies, along with their functions in the outer ER environment. NADPH, Nicotinamide adenine dinucleotide phosphate; G6P, glucose-6-phosphate; 6PG, 6-phospho gluconate; Nox4, NADPH oxidase 4; GSH, glutathione; GSSG, glutathione disulphide; ERO1, ER oxidoreductin 1; PDI, protein disulfide isomerase. CYP/p450; Cytochrome p450.

Protein disulfide isomerase (PDI) is an essential and well-characterized enzyme of disulfide bond formation in the ER. It is one of the most abundant proteins in cells and is found not only in animals, but also in plants and fungi. There are 20 oxidoreductases that are reported to catalyze disulfide bonds and are the correct sequence of pairs of cysteine residues with varying redox potential and substrate specificity [23,24]. During chaperone-assisted disulfide bond formation between polypeptide chain substrates, two electrons are provided to the cysteine residue within the PDI active site [25]. This transfer of electrons results in the reduction of the PDI active site and oxidation of the substrate. The suggested main ER-originating ROS production process and its associated ROS release mechanisms are depicted in Figure 1. Cysteine and glutathione have been previously studied in terms of maintenance of the ER redox environment [26,27]. However, a genetic study of yeast revealed a preserved ER membrane-associated protein, endoplasmic reticulum oxidoreductin-1 (ERO1) [28], which oxidizes PDI. In the pathway of oxidative proteins, electron transfer from PDI to molecular oxygen and ERO1 uses a flavin adenine dinucleotide (FAD)-dependent reaction. This electron transfer suggests that ERO1 has a strong association with protein load in the ER and can trigger ROS generation and contribute to ER stress. PDI has a chaperone effect and, along with thiol oxidoreductase, prevents protein aggregation and maintains hydrophobic pockets within the main protein core [29]. The mobile arm of PDI opens when oxidized and closes when under reduced conditions [30,31]. In the ER lumen, PDI introduces or reshuffles disulfide bonds in nascent proteins for membrane insertion or secretion [32]. PDI and other isoforms are the convergent centers of ER redox homeostasis and signaling processes [33]. In the overexpressed ERO1 model, PDI is shifted from the reduced to the oxidized state that ultimately influences its substrate. Just before ROS generation by molecular oxygen, electrons undergo several thiol-disulfide exchange reactions during disulfide bond modifications. These superoxide anion radicals help to further stimulate ROS generation. Through an estimation analysis, it has been revealed that around 25% of ROS are generated by disulfide bonds in the ER during oxidative protein folding [34]. Therefore, proteins with many disulfide bonds have a larger role in ROS generation compared to proteins with fewer such bonds.

## 3. Specific Mechanism of ER Stress-Induced ROS: NADPH Oxidase 4 (Nox4)

Nicotinamide adenine dinucleotide phosphate (NADPH) oxidase is a protein family with seven members including Nox 1–5 and Duox 1 and 2 [35]. The isoform that is most consistently associated with the ER is NADPH oxidase 4 (Nox4). Several additional locations of Nox4 have also been identified, including the mitochondria, nucleus, focal adhesions, and the cytoskeleton [36]. Moreover, a recently developed monoclonal antibody of Nox4 was localized to the plasma membrane (as sub cellular localized and the first monoclonal antibody for Nox4 in HEK293 cells). The gene that encodes Nox4 is found on chromosome 11 and is transcribed into one unspliced and 16 spliced mRNAs. Nox4 associated with p22phox uses NADH or NADPH (as an electron donor) for oxygen reduction to produce a superoxide anion [37]. Nox4 is also implicated in ROS generation; Nox4 does not require another cytosolic or membrane component for its activation. Because of the special characteristics of its C-terminal, electrons are constitutively transferred from NADPH to FAD [38]. Nox4 was shown to physically interact with PDI; in the absence of PDI, Akt phosphorylation decreased, inducing cell death [39]. The interaction between PDI and p^22phox^ was also observed in macrophages [40]; however, the downstream role of ROS generated by PDI-Nox4 could be related to ER-mediated phagocytosis, similar to PDI-ERO1.

ER stress and generation of ROS are fundamental components of the acute and chronic conditions of UPR signaling. However, ROS production itself has been shown to trigger the UPR in a few cases [41]. Similarly, after UPR activation, peripheral vasculature cells experience an increase in level of Nox4, which causes an increase in ROS generation [42]. In yeast, the ER is under oxidizing conditions during UPR [43] or is somehow opposed to the redox imbalance seen in mammalian cells, which argues against a major source of oxidants in ER stress. Under stressed conditions, Nox4 expression is increased, e.g., tunicamycin increased the expression of Nox4 10-fold [41] and increased mRNA and protein expression through 7-ketocholesterol-induced stress [44]. Under stressful conditions, the generation of ROS is also increased. Studies have revealed that silencing of Nox4 accounts for UPR-induced cellular ROS generation and is also responsible for pro-apoptotic and pro-adaptive signaling *i.e.*, chaperone expression [41]. Nox4-linked ROS generation also increases a representative oncogene, Ras level, which after activation of RhoA in the cytosol, might cause autophagy as a cellular protective mechanism. However, when Nox4/Atg5 is disabled, cells undergo apoptosis [45].

## 4. Coupled Glutathione within the ER

As explained above, during disulfide bond formation, ERO1 is oxidized by molecular oxygen, resulting in H_2_O_2_ generation and production of oxidized glutathione from glutathione (GSH) as a by-product. Both cause a disturbance in the redox status of the ER lumen, which leads to ER stress after oxidative stress [46,47]. The ratio between the oxidized and reduced forms of glutathione is an indicator of the redox environment and plays an important role in molecular mechanisms such as proliferation, differentiation, and cell death after apoptosis [48]. It is believed that glutathione is involved in PDI oxidation; however, it was previously shown that PDI oxidation is catalyzed by ER flavoprotein ERO1 both *in vitro* and *in vivo* [27,34,49].

It has been shown that GSH plays a core role in maintaining ER oxidoreductases in a reduced state in order to catalyze reduction or isomerization reactions [50,51]. It is under investigation why an oxidizing equilibrium of GSH is required in the ER lumen and how it is sustained. Moreover, under oxidative stress conditions, GSH acts as a redox buffer source, in addition to its role in disulfide bond formation. During disulfide exchange, PDI is oxidized by ERO1, which causes reduced ERO1 formation [52]. In the presence of FAD and oxygen, ERO1 can be oxidized, indicating oxygen as an electron acceptor [49]. The re-activation of ERO1 by molecular oxygen is the cause of ROS generation in the ER. The mechanism of cellular protection from an injury caused by ER-associated ROS is under investigation; however, recent data has suggested that GSH has a vital role in this process.

In the protein translocation process, reducing equivalents continuously enter as cysteine residues that undergo oxidation and reduce PDI during the disulfide bond formation process. Later, PDI oxidation by ERO1 causes ROS generation. These already-produced ROS molecules may distribute in the cytosol, interact with other organelles, and react with GSH in the ER to increase glutathione disulphide (GSSG) level. This then leads to a disturbance in the [GSH]:[GSSG] ratio.

As explained earlier, disulfide bond formation leads to the formation of ROS in both yeast and mammalian systems. ER stress in mammalian cells involves the transmembrane kinases ATF6, IRE1, and PERK [53]. In ER stress conditions, eIF2α phosphorylation by PERK reduces mRNA translation to decrease stress. Moreover, PERK stimulates the synthesis of transcription factors ATF4 and NF-E2-related factor 2 (Nrf2) [54,55]. Both of these transcription factors increase amino acid metabolism and generation of GSH [54,55]. Thus, protein synthesis decreases and GSH production increases. Further, the cells that lack PERK are more prone to experience, and be sensitive to, hydrogen peroxide and ER stress. GSH is most importantly required to increase the levels of ROS to maintain homeostasis and redox balance. Thus, the mammalian and yeast cells adapt to stress conditions and increase the synthesis of GSH to reduce ROS levels for redox balance. ROS generation is also linked with the misfolding of the protein carboxypeptidase Y (CPY*); however, studies have shown that CPY* (with mutated cysteine) overexpression does not show similar increases in ROS levels. However, after addition of glutathione to the media, cells show reduced levels of ROS and significant growth. Therefore, when mammalian and yeast cells are under stress, an increase in glutathione levels acts in a supportive way to reduce ROS and facilitate disulfide bond formation.

## 5. NADPH-Dependent p450 Reductase and p450 Connection Involvement in ER Stress

The microsomal monooxygenase (MMO) system is one of the major sources of ROS in the ER. The main function of the MMO system is to oxygenate exogenous and some endogenous substrates such as squalene monooxygenase [56], fatty acid desaturase [57], 7-dehydrocholesterol reductase, and heme oxygenase. This variety of electron-accepting partners of NADPH-p450 reductase (NPR) suggests an important role of NPR in many physiological processes. The MMO system produces ROS such as superoxide anion radicals and H_2_O_2_ [58,59]. The efficiency or degree of coupling of electron transfer from NADPH to p450 is usually <50%–60% and is often as low as 0.5%–3.0%. This “electron leakage” plays a significant part in ROS generation, while redox cycling occurs between NPR and eukaryotic p450s. ROS production is also increased by electron leakage from ER stress-associated p450 2E1 activation.

Increased cellular p450 2E1 protein activity and expression in association with enhanced ROS generation have been reported in animal models. In addition, phosphorylation of insulin receptor substrate (IRS)1/2 is controlled by JNK phosphorylation as a signal transduction mechanism [60]. It has also been suggested that elevated ROS levels impair insulin signaling through several complex mechanisms, including phosphorylation of JNK and the subsequent downstream serine/threonine phosphorylation of IRS1 [61]. p450 2E1 acts as a crucial player in ER stress-induced ROS production by inhibiting ROS through an inter-connection between NPR and p450 2E1.

Polyenylphosphatidylcholine and chlormethiazole are two inhibitors of p450 2E1 and have shown fractional but effective protection against ethanol-induced hepatic injury [62]. This fractional protection shows that p450 2E1 is not the only source of pro-oxidants. Conversely, p450 2E1 cells have shown an increase in antioxidant enzymes, such as heme-oxygenase glutathione-*S*-transferase and catalase, along with GSH level. Induction of these enzymes decreases with antioxidant treatments, indicating that p450 2E1-linked ROS governs the transcription of antioxidant genes. Although the induction of p450 2E1 protects against deleterious substances and toxins, some inhibitors of p450 2E1, such as YH439, might also inhibit other p450 family enzymes and become a source of cellular toxicity. As a result, p450 2E1 activation and expression have led to increases in ROS levels in some fatty liver diseases that further progress to steatohepatitis. A considerably higher severity of steatosis has been seen in non-alcoholic steatohepatitis compared with non-alcoholic fatty liver disease [63]. In a protective mechanism against stress, Nrf2 is also induced by p450 2E1 [64]. Although mechanism-based investigation is required, it is evident that liver injury and insulin resistance both involve p450 2E1 activation. In other studies, it has been shown that Bax inhibitor 1 (BI-1) interacts with p450 2E1 to reduce ER stress-induced ROS generation. In the presence of BI-1, the production of p450 2E1 decreases, reducing the ROS accumulation [65].

## 6. ER and Mitochondria Connection and Relationship to ROS

In earlier stages of ER stress conditions, oxidative stress forces calcium out of the ER and causes reuptake by mitochondria. Therefore, increasing the concentration of calcium increases metabolic activities and ROS generation in mitochondria. Via feedback mechanisms, calcium ions further enhance the sensitivity of calcium channels [66,67]. The release/migration of calcium is highly associated with ER stress conditions. Under ER stress, calcium release via inositol triphosphate receptors (IP_3_R) has been suggested to be induced by GSH and xanthine/xanthine oxidase [68]. 1-methyl-4-phenyl-1,2,3,6-tetrahydropyridine(MPTP) opening regulated by thiols and redox sensitivity of IP_3_R has also been reported to increase mitochondrial ROS generation, resulting in the release of ER calcium [69,70]. In the presence of ROS, ER calcium release is increased due to the action of cyclic-ADP ribose (cADPR) [71]. This may lead to more ROS generation, causing further opening of MPTP [72]. At higher concentrations, ROS can inhibit calmodulin function by suppressing ryanodine receptor (RyR). At low concentrations of ROS, calcium release is caused by activation of cADPR production, which involves calmodulin-induced RyR [73]. ROS-based sensitization involving the release of calcium has been shown to produce recurring calcium spikes due to continuous ER stress-triggered MPTP opening. ER stress has an important impact on signaling systems; if calcium in the mitochondrial matrix causes MPTP opening and rapid dissipation or cessation of ATP, mitochondrial swelling occurs. ER calcium travels toward the mitochondria and enhances metabolism, thereby increasing the supply of ATP; it also increases mitochondrial generation of ROS signaling, which cycles back to the ER, leading to further release of calcium [72].

ROS generation is also a result of calcium leakage in both ER and oxidative stress, as variation in calcium level is strongly associated with ER stress-linked ROS generation [74,75]. It is believed that ROS result from the ER-electron coupling system, *i.e.*, the PDI and ERO1α, intra ER-GSSG/GSH, or NPR system. During ER stress, calcium stores are depleted due to release from the ER. Increased level of mitochondrial calcium alter metabolism and eventually ROS production [76]. This increase in oxidative phosphorylation and ROS generation induces the mitochondria to work faster and consume more oxygen. In addition, under high mitochondrial ROS generation, as oxygen consumption increases, nitric oxide synthase is stimulated by calcium and inhibits the activity of complex IV, which further increases ROS production [77,78,79]. This signaling axis usually operates at physiological concentrations of nitric oxide (NO). At the same time, NO and high-calcium mitochondria can inhibit complex I, open MPTP to release cytochrome c to block the respiratory chain at complex III, and thus initiate ROS generation [80]. Calcium also modifies the redox environment by disrupting GSH inhibition.

This increase in ROS levels in the mitochondria triggers the ER to release calcium and sensitizes a calcium-releasing channel in the ER membrane, sending a feedback signal [81]. ER and oxidative stress have close associations in ROS generation either by induction of cytoplasmic calcium or by increasing ROS levels in the mitochondria, which then act on the ER calcium release channels.

## 7. Disease Application

ER is responsible for the synthesis and proper folding of proteins. Misfolded proteins, on the other hand, are disposed of by ERAD. Excess workload beyond the capacity of the ER causes activation of the ER stress response for cytoprotection. To decrease the burden on the ER, this response induces both chaperone and ERAD component expression. A failure of the ER stress response caused by various factors such as age, mutations, or idiosyncratic or environmental factors can result in different diseases. In this review, we summarize recent progress in the understanding of molecules that regulate the ER stress response and are potential candidates for drug targets in various conformational diseases.

### 7.1. ER Stress and Diseases

**Figure 2:**
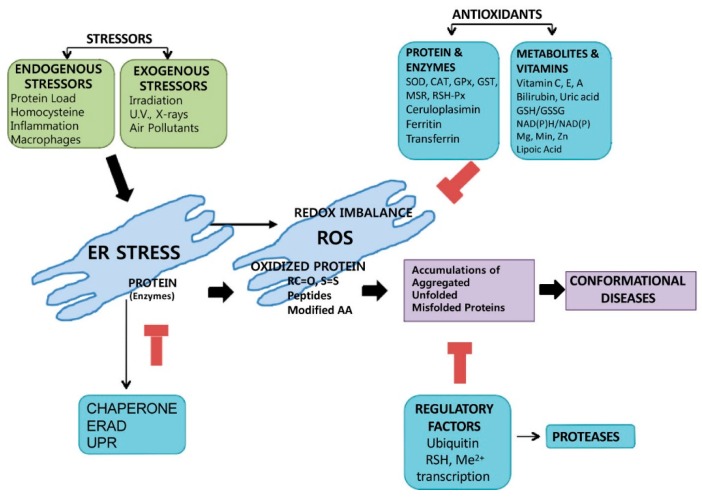
Aggregation of oxidized proteins depends on the balance of redox signaling mediators, anti-oxidants, pro oxidants, and proteolytic activities in the ER. In the presence of stressors, redox imbalance causes a protein load that leads to ER stress. Then, accumulation of oxidized proteins causes the aggregation, misfolding, or unfolding of proteins and thus the occurrence of conformational diseases. SOD, superoxide dismutase; CAT, catalase; GPx, glutathione peroxidase; GST, glutathione transferase; MSR, methionine sulfoxide reductase; RSH-Px, thiole specific peroxidase. The black arrows are showing the stimulation or the flow of mechanism, while red/T-like arrows are showing the blocking of the process.

Although unfolded protein aggregation occurs both in the ER and cytoplasm, under ER stress conditions, ROS-associated small protein aggregates are more harmful than larger ones because they bind the TATA-binding protein and cAMP response element-binding protein (CREB) (transcription factors) and impair the ubiquitin-proteasome pathway [82,83]. Conversely, larger protein aggregates and the molecular chaperones, for example, TCP1-ring complex (TRiC) and Heat Shock Protein 70 (HSP70), act in cytoprotection through further suppression of aggregate formation [84,85]. It has also been reported that Breast Cancer 1 (BRCA1, tumor suppressor) and estrogen receptors synergistically affect and contribute to Nrf2 regulation, which controls ROS levels, cell metabolism, and antioxidant activities [86]. These conformational diseases and the ER stress-associated flow process are depicted in Figure 2. Schematically, the figure suggests that the aggregation of oxidized proteins depends on the balance of redox signaling mediators, anti-oxidants, pro-oxidants, and proteolytic activity, ultimately providing the molecular mechanism of conformational diseases.

#### 7.1.1. Neurodegenerative Diseases

Parkinson’s disease (PD) is considered a neurodegenerative disorder and is characterized by failure of dopaminergic neurons in the substantia nigra with the presence of perkin, α-synculin, and intra-neuronal cytoplasmic inclusion bodies, known as Lewy bodies, in neuronal cell bodies. However, the cause of the reduction in dopaminergic neurons is still unknown. It has been suggested that neuronal cell death, along with mitochondrial dysfunction, ER stress, and oxidative stress, is involved. It is believed that the activity of ATF6 is inhibited by α-synuclein, which characteristically aggregates in PD [87]. In addition, the deficiency of nutrients, α-synuclein aggregation, and the ER stress response are induced [88], triggering chronic ER stress and leading to ROS accumulation and dopaminergic neurodegeneration [89]. The accumulation of ER stress-associated proteins, such as Hrd1p/Der3p (HRD1), that enhance ubiquitination, has been observed in neurons in the brains of PD patients [90].

In contrast, some reports have shown that the pathologic status of PD is improved by activating UPR transcription factors. In human and cell studies, a UPR-activating reagent increased UPR-target gene expression in dopaminergic neurons and decreased neuronal death in MPTP-treated conditions [91]. Consistently, gene therapy to deliver an active form of XBP1, a main regulator of UPR, has shown the potential for neuro-protection and reduction of striatal denervation in 6-hydroxydopamine-injected mice [89]. The relationship between ER stress and PD is not clear. However, the agents that modulate ER stress might be considered helpful in treating PD, as the success of pharmacological approaches including 4-phenyl butyric acid (PBA) and other ER stress regulators have been demonstrated [92,93].

Alzheimer’s disease (AD) is another devastating neurodegenerative pathological process, the most common form of dementia, and is characterized by a progressive decrease in cognitive functions, intellectual abilities, and productivity. In AD, considerable cell death and neuronal loss were reported in different areas of brain, including the neocortex and hippocampus (essential for memory and learning) [94]. Intracellular neurofibrillary tangles consist of aggregation of hyperphosphorylated tau protein and β-amyloid deposition, which cause the appearance of AD symptoms [95]. In the pathophysiology of AD, oxidative stress is involved in aging and its associated diseases; according to autopsy studies, the PERK-eIf2α pathway is hyper-activated, producing ER stress [96,97]. In AD patient brains, ER stress-associated oxidative stress has been confirmed. Specifically, PDI is highly *S*-nitrosylated in AD brains compared with controls. Thus, redox modification under ER stress might increase ROS production and is probably linked to β-amyloid aggregation [98]. The aggregation of β-amyloid in AD also enhances the ER load, ultimately inducing alteration in the morphology of both ER and mitochondria [99]. Next, the mitochondrial membrane potential dissipates, further accumulating mitochondrial ROS [100]. Agents protective against ER stress, such as glutamine and antioxidants like α-tocpopherol, ascorbic acid, and β-carotine, reduce/block ROS production [101], protecting neuronal cells against free radicals [102,103,104]. These studies strongly advocate a clear linkage between ER stress and AD. Most likely, ER stress-linked ROS is responsible for the β peptide aggregation in AD.

Prion diseases, also recognized as transmissible spongiform encephalopathies (TSEs), is another class of neurodegenerative disorders [105]. The pathophysiology of TSE involves the accumulation of conformationally altered cellular prion proteins (PrP^C^). In its normal conformation, PrP^C^is involved in cell growth, differentiation, and cell survival. Previous studies have shown the defensive role of PrP^C^ against ROS [106,107,108]. Under stress conditions, PrP^C^ expression was highly increased, and the direct interaction between ROS and PrP^C^ has been proposed in a separate study [109], which suggests that PrP^C^ plays a vital role in the removal of ROS from the extracellular environment. Thus, it is hypothesized that PrP^C^ is a preferential target of ROS in the central nervous system. Because a comparatively long postmortem delay process is required due to the risk of infection, the analysis of UPR activation markers, a particularly phosphorylated form of proteins, is difficult in prion diseases. Hetz and colleagues have reported an increase in the levels of caspase-12 activation and ER stress markers Grp78, Grp58, and Grp94 in cortical samples in prion infection [110].

Extended polyglutamine (polyQ) repeats cause genetic neurodegenerative disorders, including Huntington’s disease (HD). These disorders have been shown to include the aggregation of proteins and selective neuronal cell death *in vitro*, in transgenic animals, and in human post-mortem brain tissue [111]. The function of ER stress in polyQ diseases is deduced from investigations showing co-localization of polyQ segments with multiple chaperone proteins, such as Hsp70 and Hsp40, which are generated during ER stress conditions [112]. Another study showed that polyQ aggregates are toxic to cells. Although we knew that polyQ proteins causing neurodegenerative disorders are cytosolic-based, this study clearly showed that they also evoke ER stress and ROS by disruption of the redox environment. Thus, polyQ proteins also restrict the role of the proteasome in degradation [113,114,115]. Interestingly, polyQ-associated neurodegeneration suppression by p97 (which is a constituent of ERAD machinery) increases the breakdown of polyQ [116]. Thus, ER stress-linked ROS is involved in the progression of polyQ diseases.

Bipolar disorder (BD) is a mood disorder with recurring episodes of mania and depression [117,118]. Genetic and non-genetic factors both contribute to increasing ROS levels beyond the capacity of redox mediators in patients with BD [119]. Cellular microarray analysis was performed in twins with discordant disease perspectives, and the expression of XBP1 and BiP was noted to be decreased. However, some reports have shown polymorphisms in the promoter regions of XBP1 and BiP, which are common in BD patients [120,121,122,123], although this issue is debatable [124]. Numerous studies have reported that BD patients demonstrate considerable changes in levels of antioxidant enzymes, lipid peroxidation, and nitric oxide; however, differing results have been obtained from other researchers, challenging the reliability of these findings [125]. According to one meta-analysis, lipid peroxidation and NO level were highly increased in BD patients; however, previously reported alterations in antioxidant enzymes were shown to not be statistically significant. In a pharmacological study, mood-stabilizing drugs such as valproic acid and lithium were highly effective in BD treatment and increased the expression of BiP/Grp78, Grp94, and calreticulin (ER chaperones) [126]. In that system, we assume ROS levels would be reduced due to the enhanced ER chaperone machinery capacitance.

#### 7.1.2. Diabetes Mellitus

Diabetes Mellitus (DM) results in chronic hyperglycemic and hyperlipidemic conditions, known as important disrupters of ER homeostasis, activating irresolvable UPR signals. Oxidative and ER stress is related to DM and plays vital roles in the pathogenesis of β-cell loss and subsequent insulin resistance [127,128]. In β-cells, synthesis of glycoproteins occurs, and the workload of insulin secretion increases ER efficiency. Mice lacking PERK were shown to develop diabetes, β-cell death, and progressive suffering with hyperglycemia with aging [129]. Particularly, PERK gene mutations occur in Wolcott–Rallison Syndrome; as the ER degenerates and is unable to fold new proteins, ROS accumulation and aggregation of misfolded proteins lead to stress and cell death [130]. Elf2α is an established downstream signal of PERK; in a PERK-elf2α-associated glucose metabolism study, eIf2α knock-in mice showed more rapid β-cell depletion in the infant stage than did PERK^−/^^−^ mice [131]. Under physiological conditions, the components of UPR in β-cells act as triggers of dysfunction and apoptosis of β-cells. Chaperones that control protein folding undergo modifications due to oxidation or glycosylation and demonstrate ROS accumulation in ER stress conditions. Antioxidants, for example, mitotempo and mitoquinine, can also prevent β-cell death in glucose and glucolipotoxicity cell models of DM [117,132,133]. In DM-associated ER stress, the regulation of ROS is suggested to be a core regulator against ER stress-mediated β-cell death.

#### 7.1.3. Atherosclerosis

Atherosclerosis is a disorder involving the hardening and narrowing of arteries due to various factors such as accumulation of cholesterol, fatty compounds, cellular debris, calcium, and other substances in the inner linings of arteries. Accumulation of homocysteine is considered to be a risk factor, produced as a result of ER stress with increased expression of BiP/Grp78, Grp94, CHOP, Herp, and regulation of T-cell death-associated gene 51 (TDAG51) [134,135,136]. In contrast, in endothelial cells exposed to oxidized lipids and UPRs, including ATF4 with XBP1, were induced along with cytokine production [137]. ER stress generated by the accumulation of homocysteine may also increase cholesterol level, which promotes apoptosis of macrophages. The debris of macrophages is deposited in blood vessels and contributes to atherosclerosis [138,139,140]. Furthermore, cholesterol-mediated ER stress enhances cytokine expression in the presence of CHOP [141]. ROS generation in ER stress-exposed endothelial cells is suggested to be a contributor to atherosclerosis. In contrast, paraoxonase 2 (ER resident enzyme) reduces ROS generation [142,143]. Similarly, phenylbutyric acid (a chemical chaperone) has been widely accepted as an ER protective agent that ameliorates ER stress-mediated ROS generation [144].

#### 7.1.4. Inflammation

Inflammation is the first immune response to infection. However, the mechanisms involved in inflammation are complex, and ER stress contributes to many different kinds of inflammation. Inflammation of the CNS can produce interferon-gamma; as a result of ER stress, apoptosis of oligodendrocytes can occur [124]. PERK^+/+^ mice have shown the loss of oligodendrocytes and an increase in hypomyelination, which confirmed the protective role of the PERK pathway against interferon-gamma-induced cell death. In lungs, lipopolysaccharide-induced ER stress is associated with expression of CHOP and its subsequent ROS accumulation and cell death [145]. ER stress-linked inflammation also particularly contributes to the development of insulin resistance [146]. Anti-inflammatory drugs such as diclofenac have shown the ability to suppress ER stress [147]. It has been shown that inflammatory markers (NF-κB, nucleotide-binding domain and leucine rich repeat (NLR), NLR family pyrin domain containing 3 (NLRP3), high mobility group box-1 (HMGB1)) and NO, along with other proteins including PERK, eIF2α, caspase 9, and caspase 3, are potentially increased by aluminum [148]. Misfolded protein accumulation has been reported in inflammatory bowel disease (IBD) and many autoimmune diseases [149]. On the other hand, calcium and calcium sensing receptors reduce inflammation activation in response to NLR family pyrin domain-containing 3 activators. Calcium sensing receptors activate inflammasomes through an inositol triphosphate (IP_3_)-mediated mechanism that releases the calcium stored in the ER, resulting in accumulation of ROS. The association of inflammasome activation with ER calcium release requires further elucidation.

#### 7.1.5. Liver Disease

ER stress-mediated ROS is involved in the pathogenesis of different diseases, such as chronic viral hepatitis, alcoholic hepatic steatosis, and non-alcoholic hepatic steatosis, through increased level of homocysteine. Alcohol inhibits the enzymes (methionine synthetase and betaine-homocysteine methyltransferase) of sulfur amino acid metabolism, resulting in homocysteine accumulation. The mechanism through which alcohol causes ER stress is still under investigation [150,151,152,153,154,155]. ER stress is also involved in hepatic cancer and in human liver carcinoma by activating ATF6 and IRE1α, along with the primary ER stress chaperone, BiP/Grp78. In this manner, cells respond to the ER stress response [156,157,158]. In acute hepatic disease, the GSH level is significantly decreased, and the accumulation of ROS accelerates protein aggregation and ER stress [159]. This may increase the development of disease and reduce hepatic functional efficiency.

#### 7.1.6. Ischemia

Hypoxia-induced ER stress has been shown to be a significant cause of ischemia-associated diseases. ER stress in neuronal cells leads to brain ischemia, activating the ATF6, IRE1, and PERK pathways [160] and inducing CHOP-mediated cell death [161]. In the heart, ER stress-induced chaperone expression has been explained as a mechanism of degeneration of cardiomyocytes [162], leading to the development of ischemic heart disease. Increased expression of UPR markers and Bip/Grp78, XBP-1, and PDI has been related to myocardial infarction in mouse hearts [163]. Hypotension from cardiac arrest or arterial occlusion is closely related to tissue hypoxic and hypoglycemic conditions and is a cause of protein misfolding. ER stress and reperfusion of affected tissues also trigger ROS production under these circumstances [164,165]. It has also been reported that prevention of apoptosis by Transforming growth factor (TGF)-β, which improves myocardial function [166], and lycopene also exerts protection through anoxia-reoxygenation, reduction of endoplasmic reticulum stress, and ROS suppression [167]. A potent free radical suppressor, edaravone, protects against lipid peroxidation and ER stress-induced hypoxia by p-eIF2α and CHOP inhibition [168].

#### 7.1.7. Kidney Disease

The excessive ROS generation induced by PAT-activated ER stress is demonstrated through the increased expression of the chaperone Bip/Grp78 [169]. In addition, it has recently been reported that crocin and quercetin prevent PAT-induced apoptosis by inhibiting the ROS-mediated ER stress pathway in a human cell model [170]. Multiple factors cause ROS-mediated ER stress and lead to cell death, such as acetaminophen, which causes renal tubular injury [171] by PERK pathway activation, CHOP induction, and caspase-12 cleavage. ER stress is also involved in age-related renal fibrosis.

Due to susceptibility to ER stress, the age-linked aggregation of oxidative carbonylated Bip/Grp78 or PDI leads to ER-induced kidney dysfunction [172]. Consistently, the ER marker Bip/Grp78 was highly expressed in the samples of seven patients suffering from uromodulin-linked renal disease [173]. Oxidative stress can also be generated by aggregation of factor VIII (a protein that is deficient in the ER in hemophilia A) [47]. These findings suggest that ER stress is a central cause of chemical-provoked renal disease, and that the ER stress response is one of the securities and protective mechanisms against kidney injury.

## 8. Conclusions

Among different sub-cellular components, the ER is one of the most unique and versatile parts of the cell because of its unique oxidizing environment. Under ER-associated stress conditions, redox-signaling mediators play key roles in ROS generation. The mitochondrion is a major contributor to the synthesis of ROS with different levels of calcium in different parts of the cell. Further studies are needed to explain the ER and ROS association for a complete understanding of the pathway, and to elucidate the mechanisms of their interactions under physiological and pathological conditions. Such information is expected to help more accurately and effectively treat or prevent ER-stress associated disorders.
